# Comparative effectiveness of biologics for patients with moderate-to-severe psoriasis and special area involvement: week 12 results from the observational Psoriasis Study of Health Outcomes (PSoHO)

**DOI:** 10.3389/fmed.2023.1185523

**Published:** 2023-06-29

**Authors:** Stefano Piaserico, Elisabeth Riedl, Lev Pavlovsky, Ronald B. Vender, Can Mert, Nithi Tangsirisap, Natalie Haustrup, Gaia Gallo, Christopher Schuster, Patrick M. Brunner

**Affiliations:** ^1^Dermatology Unit, Department of Medicine, University of Padova, Padua, Italy; ^2^Department of Dermatology, Medical University of Vienna, Vienna, Austria; ^3^Department of Dermatology, Rabin Medical Center, Sackler Faculty of Medicine, Tel Aviv University, Tel Aviv, Israel; ^4^Dermatrials Research Inc. and Venderm Consulting, Hamilton, ON, Canada; ^5^HaaPACS GmbH, Schriesheim, Germany; ^6^Eli Lilly and Company, Indianapolis, IN, United States; ^7^Department of Dermatology, Icahn School of Medicine at Mount Sinai, New York, NY, United States

**Keywords:** scalp, face, palmoplantar, nails, genitalia, treatment, biologics, psoriasis

## Abstract

**Introduction:**

Psoriasis localized at the scalp, face, nails, genitalia, palms, and soles can exacerbate the disease burden. Real-world studies comparing the effectiveness of treatments for these special areas are limited.

**Methods:**

Psoriasis Study of Health Outcomes (PSoHO) is an international, prospective, non-interventional, study comparing the effectiveness of anti-interleukin (IL)-17A biologics (ixekizumab and secukinumab) compared to other approved biologics and the pairwise comparative effectiveness of ixekizumab relative to five other individual biologics for patients with moderate-to-severe psoriasis. To determine special area involvement, physicians answered binary questions at baseline and week 12. The proportion of patients who achieved special area clearance at week 12 was assessed. Missing outcome data were imputed as non-response. Comparative treatment analyses were conducted using frequentist model averaging.

**Results:**

Of the 1,978 patients included, 83.4% had at least one special area involved at baseline with the scalp (66.7%) as the most frequently affected part, followed by nails (37.9%), face/neck (36.9%), genitalia (25.6%), and palms and/or soles (22.2%). Patients with scalp, nail, or genital, but not palmoplantar or face/neck psoriasis, had significantly higher odds of achieving clearance at week 12 in the anti-IL-17A cohort compared to the other biologics cohort. Patients with scalp psoriasis had a 10–20% higher response rate and significantly greater odds (1.8–2.3) of achieving clearance at week 12 with ixekizumab compared to included biologics.

**Conclusion:**

Biologics demonstrate a high level of clearance of special areas at week 12 in a real-world setting. Patients with scalp, nail, or genital involvement have significantly higher odds of clearance at week 12 with anti-IL-17A biologics compared to other biologics.

## Introduction

Psoriasis (PsO) is a common, chronic, immune-mediated inflammatory disease that can affect all parts of the body, yet the involvement of some special areas of the body is associated with a disproportionate impact on daily functioning and quality of life (1–4). Reduction in a patient's quality of life is likely due to the associated symptoms, treatment challenges, or the visibility of psoriasis lesions in these special areas, including the scalp, face, nails, genitals, and palms and soles (5–8). However, large real-world studies that evaluate and compare the effectiveness of different treatments for PsO localized in these special areas are still limited (2, 3, 9).

The Psoriasis Study of Health Outcomes (PSoHO) is a large, international, prospective, non-interventional study that compares the effectiveness of biologics for patients with moderate-to-severe PsO (10, 11). In this study, we investigate the prevalence of special area involvement in a real-world setting and the comparative effectiveness of approved biologics for the treatment of patients with special area involvement of the scalp, genitalia, nails, face and/or neck, or palms and/or soles. We evaluate the comparative effectiveness of anti-interleukin (IL)-17A biologics compared to other approved biologics for the clearance of PsO in these special areas and provide pairwise comparative effectiveness of ixekizumab (IXE) compared to five other individual biologics (10–12).

## Methods

Details of the PSoHO study and enrolled patients have been published previously (10, 11). Briefly, the PsoHO study enrolled 1,981 adult patients from 23 countries with a confirmed diagnosis (at least 6 months before baseline) of moderate-to-severe PsO who initiated or switched biologic treatment during routine medical care (10). At baseline and week 12, physicians answered binary questions to determine special area involvement of the scalp, genitalia, nails, face, and/or neck and palms and/or soles. Prescribed biologics were grouped into the anti-IL-17A antibodies cohort [IXE and secukinumab (SEC)] and a second cohort of other biologics [brodalumab, adalimumab (ADA), certolizumab, etanercept, infliximab, ustekinumab (UST), guselkumab (GUS), risankizumab (RIS), and tildrakizumab]. Only treatment groups with more than 100 patients are shown (IXE, SEC, GUS, RIS, ADA, and UST).

Descriptive statistics and comparative effectiveness analyses using frequentist model averaging (FMA) are reported as previously published (10). Pairwise comparisons of baseline demographics between the anti-IL-17A and other biologic cohorts and IXE compared to other individual biologics were performed using Fisher's exact test or chi-square for categorical variables and analysis of variance (ANOVA) or exact *P*-value from the median test (Monte Carlo estimate) for continuous variables. For each special area, analyses were completed for patients with special area involvement at baseline and a valid result at week 12. Adjusted comparative analyses between cohorts or treatments determined the odds ratios (ORs) of patients with involvement of a specific special area at baseline who achieved complete clearance at week 12. Models were adjusted for the covariates previously described (10). Statistical significance is indicated when the 95% confidence intervals (CIs) do not cross the null hypotheses (OR = 1). Unadjusted response rates for this outcome are also reported with missing data imputed as non-response. The impact of any potential unmeasured confounding was assessed using the E-value (13).

All patients were required to give informed consent for participation in the study. The study was registered at the European Network of Centres for Pharmacoepidemiology and Pharmacovigilance (ENCEPP) (14) and was conducted according to Good Pharmacoepidemiology Practices guidelines and the Declaration of Helsinki.

## Results

**Figure 1:**
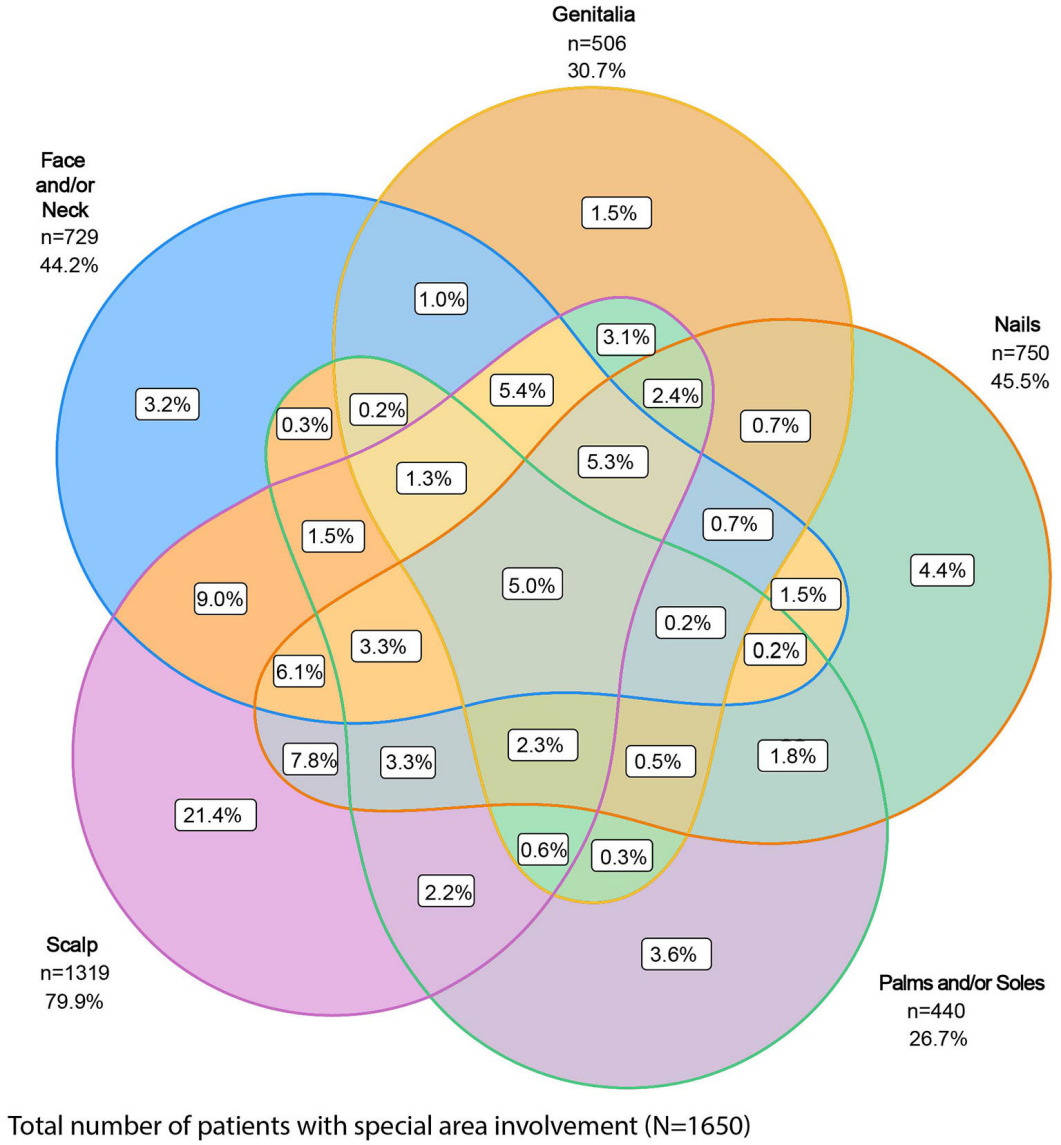
Proportion of patients with involvement of one or more special areas of psoriasis. Special areas are the scalp, genitalia, face and/or neck, nails, and palms and/or soles.

Of the 1,978 patients with special area data at baseline, 83.4% (*n* = 1,650) had at least one special area involved, with the scalp (66.7%) the most frequently affected, followed by nails (37.9%), face and/or neck (36.9%), genitalia (25.6%), and palms and/or soles (22.2%) (Table 1). Of these 1,650 patients, 66% had more than one special area involved and 5.0% had involvement in all five special areas (Figure 1).

**Table 1:** Demographics and disease characteristics of patients with psoriasis at baseline.

	**Overall (*n* = 1,981)**	**Anti-IL-17A (*n* = 773)**	**Other biologics (*n* = 1,208)**	**IXE (*n* = 532)**	**SEC (*n* = 241)**	**GUS (*n* = 303)**	**RIS (*n* = 259)**	**ADA (*n* = 284)**	**UST (*n* = 127)**
Age	45.3 (13.6)	46.8 (13.7)^*^	44.4 (13.5)	47.4 (14.1)	45.4 (12.8)	44.2 (13.2)^†^	44.1 (13.7)^‡^	45.1 (13.0)^‡^	46.4 (14.5)
Male, *n* (%)	1,143 (57.7)	442 (57.2)	701 (58.0)	313 (58.8)	129 (53.5)	179 (59.1)	161 (62.2)	163 (57.4)	77 (60.6)
Weight (kg)	85.0 (21.1)	85.6 (20.8)	84.6 (21.2)	86.3 (20.4)	83.9 (21.6)	84.0 (21.2)	83.8 (22.6)	86.7 (21.3)	82.9 (17.1)
BMI (kg/m^2^)	29.0 (6.7)	29.2 (6.6)	28.9 (6.7)	29.4 (6.6)	28.9 (6.5)	29.0 (6.7)	28.6 (6.9)	29.3 (6.6)	28.0 (5.6)^‡^
White race, *n* (%)	1,441 (72.7)	576 (74.5)	865 (71.6)	394 (74.1)	182 (75.5)	162 (53.5)^†^	169 (65.3)^‡^	248 (87.3)^†^	99 (78.0)
Disease duration, median years (Q1, Q3)	14.0 (6.8, 23.8)	14.3 (6.4, 24.2)	13.8 (7.1, 23.6)	13.9 (6.7, 25.3)	14.9 (6.0, 21.8)	14.9 (7.8, 24.4)	13.7 (8.2, 23.5)	14.2 (6.3, 25.0)	12.1 (6.3, 23.7)
PASI	14.5 (8.6)	14.6 (8.5)	14.5 (8.6)	14.4 (8.5)	15.0 (8.7)	14.6 (9.3)	15.4 (9.8)	13.3 (7.1)	14.4 (7.9)
Percentage of BSA	21.3 (17.7)	21.1 (17.5)	21.5 (17.9)	20.6 (17.2)	22.3 (18.1)	21.7 (18.5)	20.6 (18.9)	20.6 (16.6)	22.6 (17.7)
DLQI^a^	12.6 (7.8)	12.9 (7.9)	12.4 (7.8)	12.6 (7.9)	13.5 (7.7)	12.3 (8.1)	11.8 (7.3)	12.9 (7.6)	12.3 (8.0)
sPGA, *n* (%)									
Moderate	988 (50.7)	387 (50.7)	601 (50.8)	267 (50.6)	120 (50.8)	143 (47.7)	102 (40.8)^‡^	170 (60.5)^‡^	68 (54.8)
Severe	610 (31.3)	242 (31.7)	368 (31.1)	176 (33.3)	66 (28.0)	101 (33.7)	93 (37.2)	69 (24.6)^‡^	37 (29.8)
Very severe	76 (3.9)	34 (4.5)	42 (3.5)	16 (3.0)	18 (7.6)^‡^	14 (4.7)	15 (6.0)	5 (1.8)	2 (1.6)
**Number of patients with baseline data for special area involvement** ^**b**^	**1,978**	**773**	**1,205**	**532**	**241**	**302**	**258**	**284**	**126**
≥1 special area involvement, *n* (%)^c^	1,650 (83.4)	638 (82.5)	1,012 (84.0)	441 (82.9)	197 (81.7)	256 (84.8)	210 (81.4)	230 (81.0)	107 (84.9)
Scalp, *n* (%)	1,319 (66.7)	494 (63.9)^**^	825 (68.5)	347 (65.2)	147 (61.0%)	207 (68.5)	172 (66.7)	187 (65.8)	82 (65.1)
Genitalia, *n* (%)	506 (25.6)	205 (26.5)	301 (25.0)	154 (28.9)	51 (21.2)^‡^	82 (27.2)	47 (18.2)^‡^	73 (25.7)	31 (24.6)
Nails, *n* (%)	750 (37.9)	305 (39.5)	445 (36.9)	221 (41.5)	84 (34.9)	115 (38.1)	88 (34.1)	105 (37.0)	45 (35.7)
Face and/or neck, *n* (%)	729 (36.9)	261 (33.8)^**^	468 (38.8)	188 (35.3)	73 (30.3)	141 (46.7)^‡^	102 (39.5)	84 (29.6)	41 (32.5)
Palms and/or soles, *n* (%)	440 (22.2)	174 (22.5)	266 (22.1)	131 (24.6)	43 (17.8)^‡^	65 (21.5)	58 (22.5)	52 (18.3)^‡^	19 (15.1)^‡^

All results are presented as the mean (standard deviation) of all available data for that measure unless otherwise stated. The anti-IL-17A cohort includes IXE- and SEC-treated patients. In cases of *n* (%) not matching the total in the group, the remainder is attributable to missing data for patients. Pairwise comparisons performed using Fisher's exact test or chi-square for categorical variables and analysis of variance (ANOVA) or exact *P*-value from the median test (Monte Carlo estimate) for continuous variables. ADA, Adalimumab; BMI, body mass index; BSA, Body Surface Area; DLQI, Dermatology Life Quality Index; GUS, Guselkumab; IXE, Ixekizumab; PASI, Psoriasis Area and Severity Index; PSSD, Psoriasis Symptoms and Signs Diary; sPGA, Static Physician Global Assessment; Q, Quartile; RIS, Risankizumab; SEC, Secukinumab; UST, Ustekinumab.

^a^DLQI was measured on a 0–30 scale.

^b^Information about special area involvement missing for three patients. Special area involvement was recorded as a yes/no question (investigator assessed).

^c^At least one special area of the scalp, genitalia, face and/or neck, palm and/or soles, or nails involved. Green shading signifies significant differences (*P*-value < 0.05) between anti-IL-17A biologics vs. other biologics.

^*^P < 0.001 vs. the other biologics cohort.

^**^P < 0.05 vs. the other biologics cohort. Yellow shading signifies a significant difference (*P*-value < 0.05) compared to IXE (shaded in yellow).

^†^*P* ≤ 0.001 vs. IXE.

^‡^*P* < 0.05 vs. IXE.

**Figure 2:**
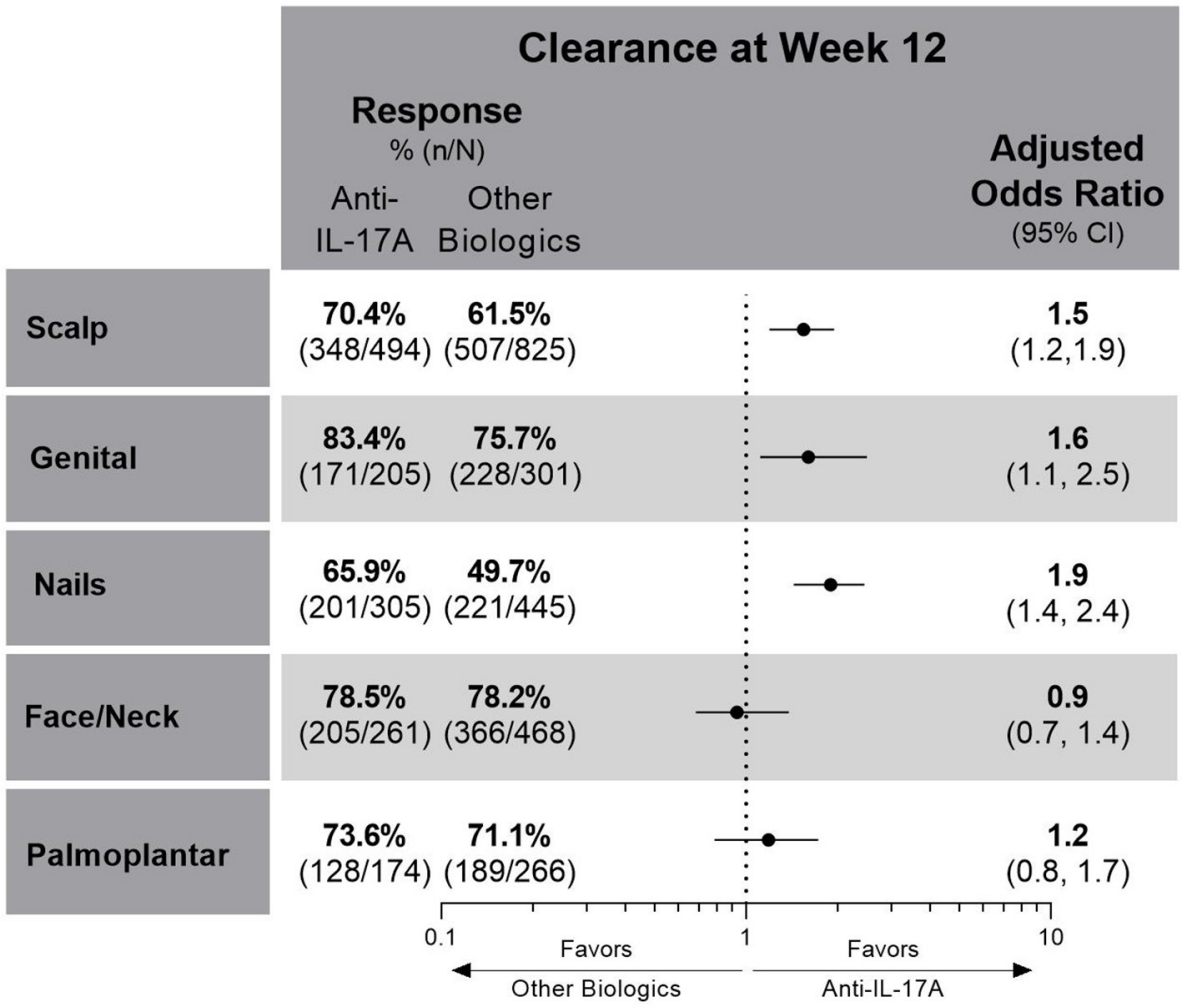
Unadjusted response rates and comparative adjusted odds ratios for the anti-IL-17A cohort compared to the other biologics cohort for patients with scalp, genital, nail, face and/or neck or palmoplantar involvement at baseline and with complete clearance of these special areas at week 12. Comparative results are statistically significant if 95% CIs of the odds ratios do not cover 1. Missing data imputed as non-response. CI, confidence interval; IL, interleukin.

Compared to the other biologics cohort, the anti-IL-17A cohort had higher unadjusted response rates and at least 50% greater odds of achieving clearance of scalp (OR 1.5; CIs 1.2, 1.9), genital (OR 1.6; CIs 1.1, 2.5), or nail (OR 1.9; CIs 1.4, 2.4) psoriasis at week 12 (Figure 2). No significant differences between cohorts were determined for patients with either face and/or neck or palmoplantar involvement, although slightly higher unadjusted response rates for clearance of these areas were achieved in the anti-IL-17A cohort compared to the other biologics cohort. In patients who received the EMA-approved on-label dosing, treatment results for special area clearance were comparable to those of the entire patient cohort (Supplementary Figure S1).

For patients with scalp involvement, the E-value for scalp clearance for the comparison of the anti-IL-17A cohort with the other biologics cohort was 1.75 [FMA OR (95% CI) = 1.5 (1.2, 1.9)], and the E-value for the lower confidence limit of the point estimate was 1.42. This E-value analysis indicated no substantial confounding (a risk ratio association of >1.75 for both the treatment selection and outcomes would be required to impact the observed treatment estimate).

**Figure 3:**
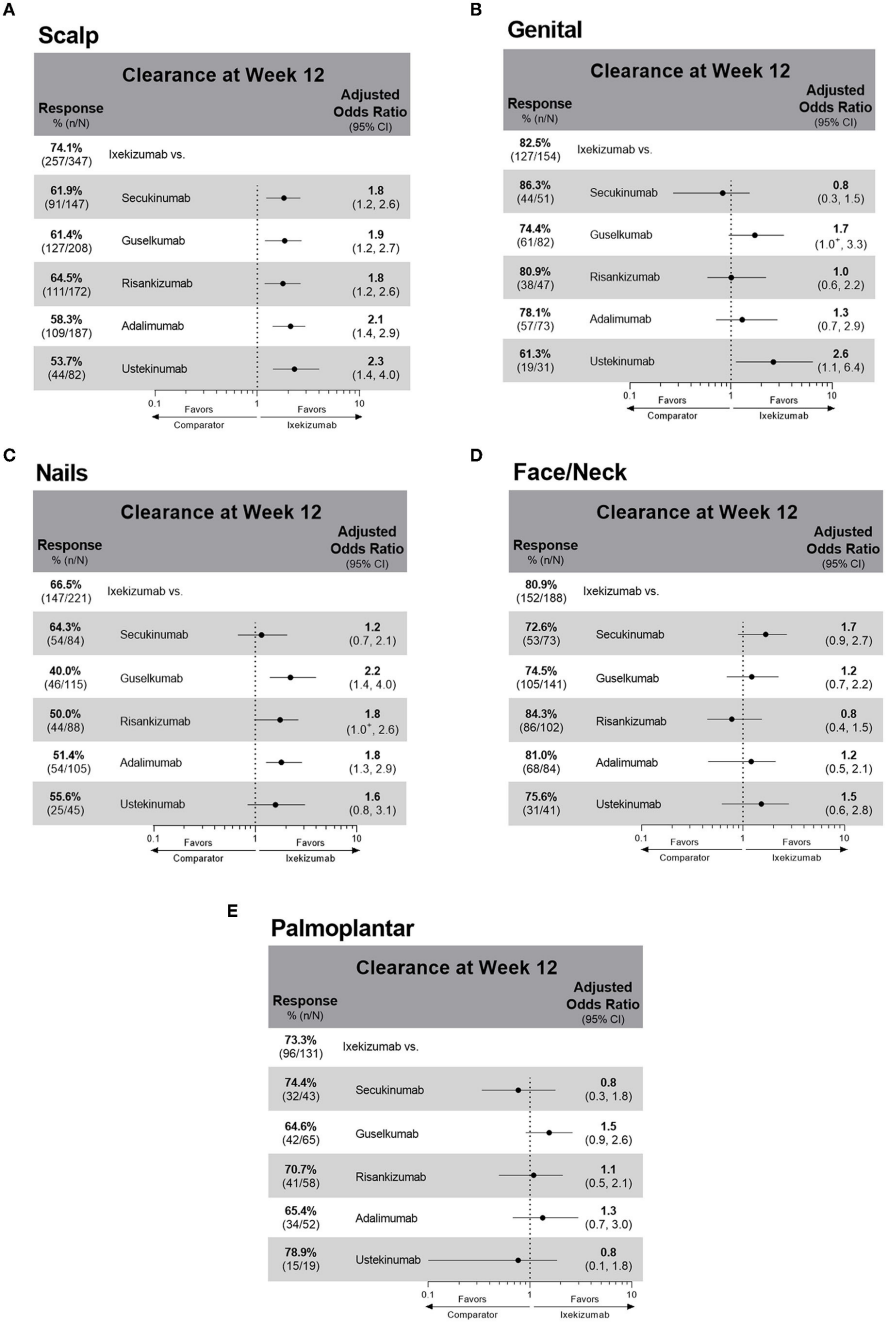
Unadjusted response rates and comparative adjusted odds ratios of ixekizumab versus individual treatments for patients with baseline involvement and clearance at week 12 of **(A)** scalp psoriasis **(B)** genital psoriasis **(C)** nail psoriasis **(D)** face and/or neck psoriasis **(E)** palmoplantar psoriasis. Comparative results are statistically significant if 95% CIs of the odds ratios do not cover 1. ^+^Denotes that the lower CI is < 1.0: The lower CI for the ixekizumab compared to guselkumab odds ratio for genital clearance is 0.952. The lower CI for the ixekizumab compared to risankizumab odds ratio for nail clearance is 0.977. Missing outcome data imputed as non-response. CI, confidence interval.

At week 12, IXE-treated patients had a higher unadjusted response rate (74%) for scalp psoriasis clearance compared to patients treated with all other studied biologics (54–65%) (Figure 3A) (Supplementary Table 1). Moreover, patients treated with IXE had 1.8–2.3 higher odds of achieving scalp psoriasis clearance at week 12 than patients treated with any of the other comparator biologics. For patients with genital involvement, treatment with SEC, IXE, and RIS resulted in unadjusted response rates of over 80% (Figure 3B). Significantly, IXE also had 2.6 times higher odds of genital psoriasis resolution at week 12 compared with UST. The greatest variability in unadjusted response rates for biologics was shown for the resolution of nail involvement (40–67%) with the highest response rate shown with IXE (Figure 3C). IXE-treated patients also had significantly higher odds of nail clearance at week 12 than GUS and ADA. No statistically significant differences in comparative effectiveness were observed between IXE and other treatments for the clearance of face and/or neck or palmoplantar involvement (Figures 3D, E). All biological treatments resulted in a high proportion of patients with clearance of face and/or neck involvement (73–84%) at week 12, but lower unadjusted response rates were reported for patients with palmoplantar (65–79%) involvement. In patients who received the EMA-approved on-label dosing (1,764/1,978; 89.2%), treatment results for special area clearance were comparable with those of the entire patient cohort (Supplementary Table 2), with the exception that ixekizumab-treated patients had significantly higher odds of nail clearance than risankizumab-treated patients (OR 2.0; CIs 1.1, 3.3; Supplementary Figure S2C).

## Discussion

In this real-world study population of 1,978 patients with moderate-to-severe PsO, the involvement of one or more special areas was prevalent. This aligns with other studies showing that PsO in one special area can increase the likelihood of involvement of other special areas, as well as for more severe disease (3, 5, 9, 15). In PSoHO, anti-IL-17A biologics show significantly greater effectiveness for scalp, genital, or nail psoriasis clearance compared with other included biologics in real-world clinical practice. The anti-IL-17A cohort also shows numerically higher response rates for clearance of all special areas at week 12 compared with the other biologics cohort. Since lack of effectiveness for special areas is one of the main reasons that patients report non-compliance with topical treatments (16), knowing the comparative effectiveness of biologics in clearing various special areas can help to inform treatment decisions. The data presented here confirm the effectiveness of anti-IL-17A biologics (10, 11, 17, 18) and extend this result to PsO in special areas of the body that are regarded as burdensome and sometimes difficult to treat.

The PSoHO study shows that approximately two-thirds of patients with special area involvement have more than one special area involved. This aligns with other studies showing that PsO in one of these special areas can be a risk factor with an increased likelihood of having the involvement of other special areas, as well as for more severe disease (3, 5, 9, 15). Scalp psoriasis was the most common special area for patients in PSoHO (66.7%), which reflects other real-world studies that record a prevalence ranging from 38 to 65% (3, 5, 9, 19). Patients with scalp involvement report greater disease and itch severity compared with those without scalp involvement (3, 7). Topical treatments are often the first option for treatment, even though the presence of hair makes the scalp less accessible, even for foams and solutions (2, 19). However, data from this study highlight the effectiveness of anti-IL-17A biologics, and, in particular, IXE at week 12 for the treatment of scalp psoriasis. Higher response rates and significantly higher odds of scalp clearance at week 12 were achieved with anti-IL-17A biologics compared with the other biologics. With more than 74% of patients achieving scalp psoriasis clearance, IXE-treated patients had a higher unadjusted response rate compared to SEC (62%), GUS (61%), RIS (65%), ADA (58%), and UST (54%) and significantly greater odds (1.8–2.3) of achieving scalp psoriasis clearance at week 12. These results confirm primary PSoHO data (10) and extend them to patients with scalp psoriasis.

More than a quarter of patients in the PSoHO study reported the presence of genital psoriasis, which is within the range of previous reports (20). Approximately 29–63% of patients with PsO are impacted by genital psoriasis at some point during the course of their disease (16, 20–22). However, genital psoriasis remains significantly underdiagnosed, with one study reporting 60% of patients with PsO were never examined in the genital area by their dermatologist (23). Furthermore, the burden of genital psoriasis is profound and has a significant impact on sexual health resulting in greater stigmatization and lower self-esteem than visible special areas (20, 24, 25). For the treatment of genital psoriasis, patients had significantly higher odds of clearance in the anti-IL-17A cohort compared to the other biologics cohort. This result also reflects that SEC and IXE treatment led to the highest proportion of patients with resolution of genital psoriasis at week 12. These results support other recent studies showing the rapid resolution of genital psoriasis with IXE (22, 26, 27).

The prevalence of nail psoriasis varies widely in the literature from 10 to 82% (28) but was reported for over a third of patients in PSoHO. Compared with other special areas, the management of nail psoriasis is particularly challenging (29, 30). This was reflected in the PSoHO data as treatment of nail psoriasis resulted in the greatest variability in response rates across biologics. Nevertheless, patients treated with anti-IL-17A biologics had significantly higher odds of clearance at week 12 compared with other biologics. IXE had 2–27% higher response rates than other individual biologics (40–64%), and IXE-treated patients had significantly higher odds of achieving clearance than GUS and ADA. These data mirror the IXORA-R and SPIRIT-H2H clinical trials data, whereby IXE demonstrated superior efficacy compared to GUS, as well as ADA, in the resolution of nail psoriasis at week 24 (31, 32). However, the use of binary questions gives rise to substantially higher unadjusted response rates than those expected using more formal assessments, such as the modified nail psoriasis severity index (mNAPSI) (33). Additionally, it would be premature to make a final assessment of nail psoriasis at 12 weeks, as longer periods are required for the nail plate to grow out and for treatment effectiveness to be evaluated. This is exemplified by one study, in which differences in treatment effectiveness between IXE and UST only emerge beyond 12 weeks (34). As such, it is prudent to wait for longer-term PSoHO results that will also include specific assessments of nail psoriasis, such as the mNAPSI.

Facial psoriasis was previously considered to be uncommon, yet in line with other studies (5, 35), PSoHO shows over a third of patients have psoriasis in this special area. Compared to other body areas that may be hidden more easily, people with facial psoriasis often feel stigmatized, which can result in isolation, depression, and reduced quality of life (36, 37). In PSoHO, there was a consistently high proportion of patients (>70%) who achieved clearance of facial psoriasis at week 12 irrespective of the biologics used, with the highest response rates with RIS, ADA, and IXE. Similar to other studies, 22.2% of PSoHO patients had palmoplantar involvement, which, together with nail psoriasis, is arguably the most difficult-to-treat special area (5, 38). Patients with palmoplantar psoriasis report greater physical disability, pain, fatigue, and lower quality-of-life scores than those without palmoplantar involvement (3, 39). Interestingly, no significant differences between treatments were found, though unadjusted response rates for palmoplantar psoriasis clearance were numerically the highest for UST, SEC, and IXE.

Observational studies have inherent limitations, including measured and unmeasured confounding bias compared with randomized clinical trials. However, the application of FMA can accommodate some of these uncertainties in model choice through the machine learning framework. The statistical precision of these comparative analyses was constrained by the number of representative patients with involvement of each special area and the respective covariates used. Limitations of this study include the grouping of non-anti-IL-17A biologics into a single category, the use of binary questions without corresponding scores, such as palmoplantar PASI (PPASI), psoriasis scalp severity index (PSSI) or mNAPSI, and the relatively short follow-up period of 12 weeks. Longer treatment periods may be necessary to fully assess and conclude the comparative effectiveness of the biologics included. Additionally, some special areas may also be challenging for the physician to differentiate, such as between the face and the scalp, which may result in overlap. It is also not possible to exclude the possibility that patients used topical treatments in addition to biologics and remains to be investigated.

This study contributes to our understanding of the treatment in these special areas by providing the comparative effectiveness of different biologics for achieving clearance of special areas after 12 weeks. In general, biologics demonstrate a high level of clearance of these special areas at week 12 in a real-world setting. In particular, patients with scalp, nail, or genital, but not palmoplantar or face and neck, involvement have significantly higher odds of achieving clearance of these areas at week 12 with anti-IL-17A biologics compared with other biologics.

## Data availability statement

The original contributions presented in the study are included in the article/Supplementary material, further inquiries can be directed to the corresponding author.

## Ethics statement

The studies involving human participants were reviewed and approved by all of the necessary central or local IRB and/or Ethics Committee approvals have been obtained for this multi-site, international study by United BioSource LLC (UBC). The patients/participants provided their written informed consent to participate in this study.

## Author contributions

CS and ER were involved with the conception and design of the work. CS, NH, and CM carried out the analysis of data. SP, ER, LP, RV, NT, NH, GG, CS, CM, and PB were involved with the interpretation of data for the work. NH, CS, and CM drafted the work. All authors contributed to the critical revision of the manuscript and approved the submitted version.

## References

[1] Sarma N. Evidence and suggested therapeutic approach in psoriasis of difficult-to-treat areas: palmoplantar psoriasis, nail psoriasis, scalp psoriasis, and intertriginous psoriasis. Indian J Dermatol. (2017) 62:113. 10.4103/ijd.IJD_539_16 28400628 PMC5363132

[2] Wozel G. Psoriasis treatment in difficult locations: scalp, nails, and intertriginous areas. Clin Dermatol. (2008) 26:448–59. 10.1016/j.clindermatol.2007.10.026 18755363

[3] Duffin KC Mason MA Gordon K Harrison RW Crabtree MM Guana A. Characterization of patients with psoriasis in challenging-to-treat body areas in the corrona psoriasis registry. Dermatology. (2021) 237:46–55. 10.1159/000504841 31962340 PMC7845438

[4] Merola JF Qureshi A Husni ME. Underdiagnosed and undertreated psoriasis: nuances of treating psoriasis affecting the scalp, face, intertriginous areas, genitals, hands, feet, and nails. Dermatol Ther. (2018) 31:e12589. 10.1111/dth.12589 29512290 PMC6901032

[5] Egeberg A See K Garrelts A Burge R. Epidemiology of psoriasis in hard-to-treat body locations: data from the Danish skin cohort. BMC Dermatol. (2020) 20:3. 10.1186/s12895-020-00099-7 32434510 PMC7238562

[6] Janowski K Steuden S Bogaczewicz J. Clinical and psychological characteristics of patients with psoriasis reporting various frequencies of pruritus. Int J Dermatol. (2014) 53:820–9. 10.1111/ijd.12074 24261840

[7] Duffin K Karki C Mason M Gordon K Har-rison R Guana A. Describing the clinical and patient-reported outcomes of patients with scalp psoriasis enrolled in the Corrona Psoriasis Registry. J Am Acad Dermatol. (2018) 79:AB105.

[8] Smith CH Yiu ZZN Bale T Burden AD Coates LC Edwards W. British Association of Dermatologists guidelines for biologic therapy for psoriasis 2020: a rapid update. Br J Dermatol. (2020) 183:628–37. 10.1111/bjd.19039 32189327

[9] Augustin M Sommer R Kirsten N Danckworth A Radtke MA Reich K. Topology of psoriasis in routine care: results from high-resolution analysis of 2009 patients. Br J Dermatol. (2019) 181:358–65. 10.1111/bjd.17403 30430557

[10] Pinter A Puig L Schäkel K Reich A Zaheri S Costanzo A. Comparative effectiveness of biologics in clinical practice: week 12 primary outcomes from an International observational psoriasis study of health outcomes (PSoHO). J Eur Acad Dermatol Venereol. (2022) 36, 2087–100. 10.1111/jdv.18376 35766124

[11] Lynde C Riedl E Maul J-T Torres T Pinter A Fabbrocini G. Comparative effectiveness of biologics across subgroups of patients with moderate-to-severe plaque psoriasis: results at week 12 from the PSoHO Study in a real-world setting. Adv Ther. (2022) 40:869–86. 10.1007/s12325-022-02379-9 36515803 PMC9988734

[12] Reich A Pinter A Maul J-T Vender RB Torres T Brnabic A. Speed of clinical improvement in the real-world setting from patient-reported psoriasis symptoms and signs diary (PSSD): secondary outcomes from the Psoriasis Study of Health Outcomes (PSoHO) Through 12 Weeks. J Eur Acad Dermatol Venereol. (2023) 1–16. 10.1111/jdv.19161. [Epub ahead of print]. 37147855

[13] VanderWeele TJ Ding P. Sensitivity analysis in observational research: introducing the E-value. Ann Intern Med. (2017) 167:268–74. 10.7326/M16-2607 28693043

[14] European Medicines Agency. Psoriasis study of health outcomes – an international observational study of 3 year health outcomes in the biologic treatment of moderate to severe plaque psoriasis. In: Pharmacovigilance ENoCfPa, editor (2018). Available online at: https://www.encepp.eu/encepp/viewResource.htm?id=25115 (accessed April 13, 2023).

[15] Strober B Ryan C van de Kerkhof P van der Walt J Kimball AB Barker J. Recategorization of psoriasis severity: Delphi consensus from the International Psoriasis Council. J Am Acad Dermatol. (2020) 82:117–22. 10.1016/j.jaad.2019.08.026 31425723

[16] Fouere S Adjadj L Pawin H. How patients experience psoriasis: results from a European survey. J Eur Acad Dermatol Venereol. (2005) 19:2–6. 10.1111/j.1468-3083.2005.01329.x 16274404

[17] Warren RB See K Burge R Zhang Y Brnabic A Gallo G. Rapid response of biologic treatments of moderate-to-severe plaque psoriasis: a comprehensive investigation using Bayesian and frequentist network meta-analyses. Dermatol Ther. (2020) 10:73–86. 10.1007/s13555-019-00337-y 31686337 PMC6994587

[18] Tada Y Rei W Hisashi N Yasumasa K Takanobu N and Kenji K. Short-term effectiveness of biologics in patients with moderate-to-severe plaque psoriasis: A systematic review and network meta-analysis. J Dermatol Sci. (2020) 99:53–61. 10.1016/j.jdermsci.2020.06.003 32600737

[19] Chan CS Van Voorhees AS Lebwohl MG Korman NJ Young M Bruce BF Jr. Treatment of severe scalp psoriasis: from the Medical Board of the National Psoriasis Foundation. J Am Acad Dermatol. (2009) 60:962–71. 10.1016/j.jaad.2008.11.890 19375191

[20] Ryan C Sadlier M De Vol E Patel M Lloyd AA Day A. Genital psoriasis is associated with significant impairment in quality of life and sexual functioning. J Am Acad Dermatol. (2015) 72:978–83. 10.1016/j.jaad.2015.02.1127 25824273

[21] Meeuwis KAP Bleakman AP van de Kerkhof PCM Dutronc Y Henneges C Kornberg LJ. Prevalence of genital psoriasis in patients with psoriasis. J Dermatol Treat. (2018) 29:754–60. 10.1080/09546634.2018.1453125 29565190

[22] Merola JF Ghislain P-D Dauendorffer JN Bleakman AP Brnabic AJM Burge R. Ixekizumab improves secondary lesional signs, pain and sexual health in patients with moderate-to-severe genital psoriasis. J Eur Acad Dermatol Venereol. (2020) 34:1257–62. 10.1111/jdv.16181 31919919 PMC7318177

[23] Gorrepati PL Argobi Y Alora MB Smith GP. Provider evaluation and patient experience among patients with genital psoriasis. Dermatol Ther. (2021) 34:e14783. 10.1111/dth.14783 33455053

[24] Schmid-Ott G Kuensebeck HW Jaeger B Werfel T Frahm K Ruitman J. Validity study for the stigmatization experience in atopic dermatitis and psoriatic patients. Acta Dermatovenereol. (1999) 79:443–7. 10.1080/000155599750009870 10598757

[25] Cather JC Ryan C Meeuwis K Meeuwis K Potts Bleakman AJ Naegeli AN. Patients' perspectives on the impact of genital psoriasis: a qualitative study. Dermatol Therapy. (2017) 7:447–61. 10.1007/s13555-017-0204-3 29076000 PMC5698203

[26] Yeh C-P Huang Y-W Tsai T-F. Comparison of the relative efficacy of different biologics in different body areas in patients with moderate to severe psoriasis receiving biologics and tofacitinib in phase 3 randomized controlled trials: a 15-year single-center experience. Expert Rev Clin Pharmacol. (2022) 15:887–95. 10.1080/17512433.2022.2103538 35848067

[27] Sotiriou E Bakirtzi K Papadimitriou I Tsentemeidou A Eftychidou P Eleftheriadis V. A head-to-head comparison of risankizumab and ixekizumab for genital psoriasis: a real-life, 24-week, prospective study. J Eur Acad Dermatol Venereol. (2022) 36:e359–61. 10.1111/jdv.17880 34923693

[28] Rigopoulos D Baran R Chiheb S III Daniel CR Di Chiacchio N Gregoriou S. Recommendations for the definition, evaluation, and treatment of nail psoriasis in adult patients with no or mild skin psoriasis: a dermatologist and nail expert group consensus. J Am Acad Dermatol. (2019) 81:228–40. 10.1016/j.jaad.2019.01.072 30731172

[29] Augustin M Reich K Blome C Schäfer I Laass A Radtke MA. Nail psoriasis in Germany: epidemiology and burden of disease. Br J Dermatol. (2010) 163:580–5. 10.1111/j.1365-2133.2010.09831.x 20456340

[30] Caputo V Strafella C Termine A Dattola A Mazzilli S Lanna C. Overview of the molecular determinants contributing to the expression of Psoriasis and Psoriatic Arthritis phenotypes. J Cell Mol Med. (2020) 24:13554–63. 10.1111/jcmm.15742 33128843 PMC7754002

[31] Blauvelt A Papp K Gottlieb A Jarell A Reich K Maari C. Leonardi C, Elewski B, et al. A head-to-head comparison of ixekizumab vs guselkumab in patients with moderate-to-severe plaque psoriasis: 24-week efficacy and safety results from a randomized, double-blinded trial^*^*. Br J Dermatol*. (2021) 184:1047–58. 10.1111/bjd.19509 32880909 PMC8246960

[32] Reich K Kristensen LE Smith SD Rich P Sapin C Leage SL. Efficacy and safety of ixekizumab versus adalimumab in biologic-naïve patients with active psoriatic arthritis and moderate-to-severe psoriasis: 52-week results from the randomized SPIRIT-H2H Trial. Dermatol Pract Concept. (2022) 12:e2022104. 10.5826/dpc.1202a104 35646453 PMC9116563

[33] Reich K Conrad C Kristensen LE Smith SD Puig L Rich P. Network meta-analysis comparing the efficacy of biologic treatments for achieving complete resolution of nail psoriasis. J Dermatol Treat. (2022) 33:1652–60. 10.1080/09546634.2021.1892024 33641593

[34] Reich K Pinter A Lacour JP Ferrandiz C Micali G French LE. Comparison of ixekizumab with ustekinumab in moderate-to-severe psoriasis: 24-week results from IXORA-S, a phase III study. Br J Dermatol. (2017) 177:1014–23. 10.1111/bjd.15666 28542874

[35] Mallbris L Larsson P Bergqvist S Vingård E Granath F Ståhle M. Psoriasis phenotype at disease onset: clinical characterization of 400 adult cases. J Investig Dermatol. (2005) 124:499–504. 10.1111/j.0022-202X.2004.23611.x 15737189

[36] Alpsoy E Polat M FettahlioGlu-Karaman B Karadag AS Kartal-Durmazlar P YalCın B. Internalized stigma in psoriasis: a multicenter study. J Dermatol. (2017) 44:885–91. 10.1111/1346-8138.13841 28407292

[37] van de Kerkhof PCM Murphy GM Austad J Ljungberg A Cambazard F Duvold LB. Psoriasis of the face and flexures. J Dermatol Treat. (2007) 18:351–60. 10.1080/09546630701341949 17907013

[38] Chung J Callis Duffin K Takeshita J Shin DB Krueger GG Robertson AD. Palmoplantar psoriasis is associated with greater impairment of health-related quality of life compared with moderate to severe plaque psoriasis. J Am Acad Dermatol. (2014) 71:623–32. 24894455 10.1016/j.jaad.2014.04.063 PMC4165651

[39] Pettey AA Balkrishnan R Rapp SR Fleischer AB and Feldman SR. Patients with palmoplantar psoriasis have more physical disability and discomfort than patients with other forms of psoriasis: implications for clinical practice. J Am Acad Dermatol. (2003) 49:271–5. 12894076 10.1067/s0190-9622(03)01479-8

