# Supplementary material for: Comparative effectiveness of biologics for patients with moderate-to-severe psoriasis and special area involvement: week 12 results from the observational Psoriasis Study of Health Outcomes (PSoHO)
Source: Front Med (Lausanne). 2023 Jun 29;10:1185523. doi: 10.3389/fmed.2023.1185523 (PMC10339811; doi:10.3389/fmed.2023.1185523)
Supplement: Supplementary file 1 [file Data_Sheet_1.docx]

**Supplementary Materials**

for

Comparative effectiveness of biologics for patients with moderate-to-severe psoriasis and special area involvement: week 12 results from the observational Psoriasis Study of Health Outcomes (PSoHO)


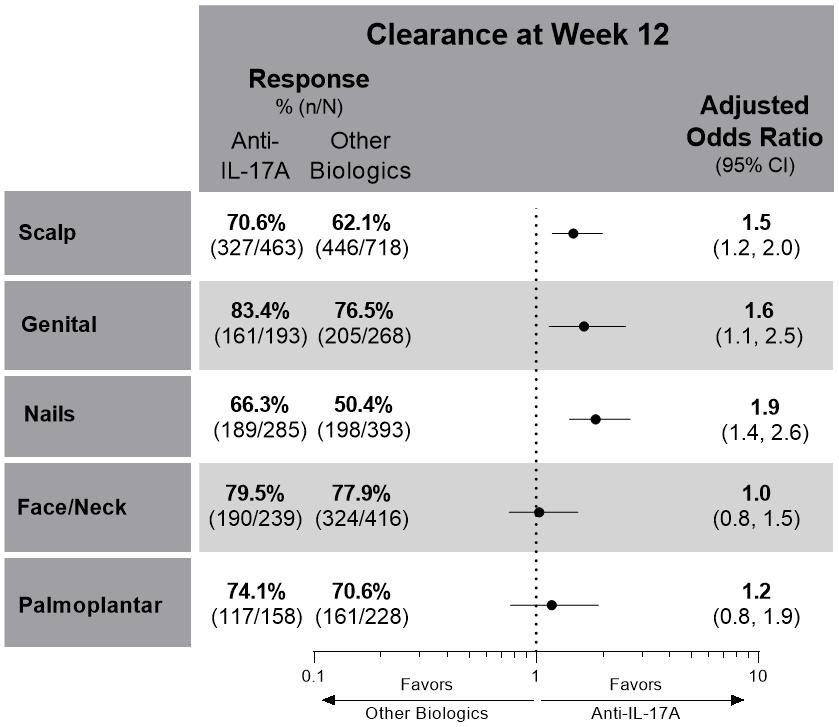


**Figure S1:** **Subgroup of patients who received the EMA-approved on-label dosing**. Unadjusted response rates and comparative adjusted odds ratios for the anti-IL-17A cohort versus the other biologics cohort for patients with scalp, genital, nail, face and/or neck and palmoplantar involvement at baseline who have complete clearance of these special areas at Week 12. Comparative results are statistically significant if 95% CIs of the odds ratios do not cover 1. Missing data imputed as non-response. CI, confidence interval; IL, interleukin n, number of patients with clearance of special area; N, number of patients in treatment cohort.


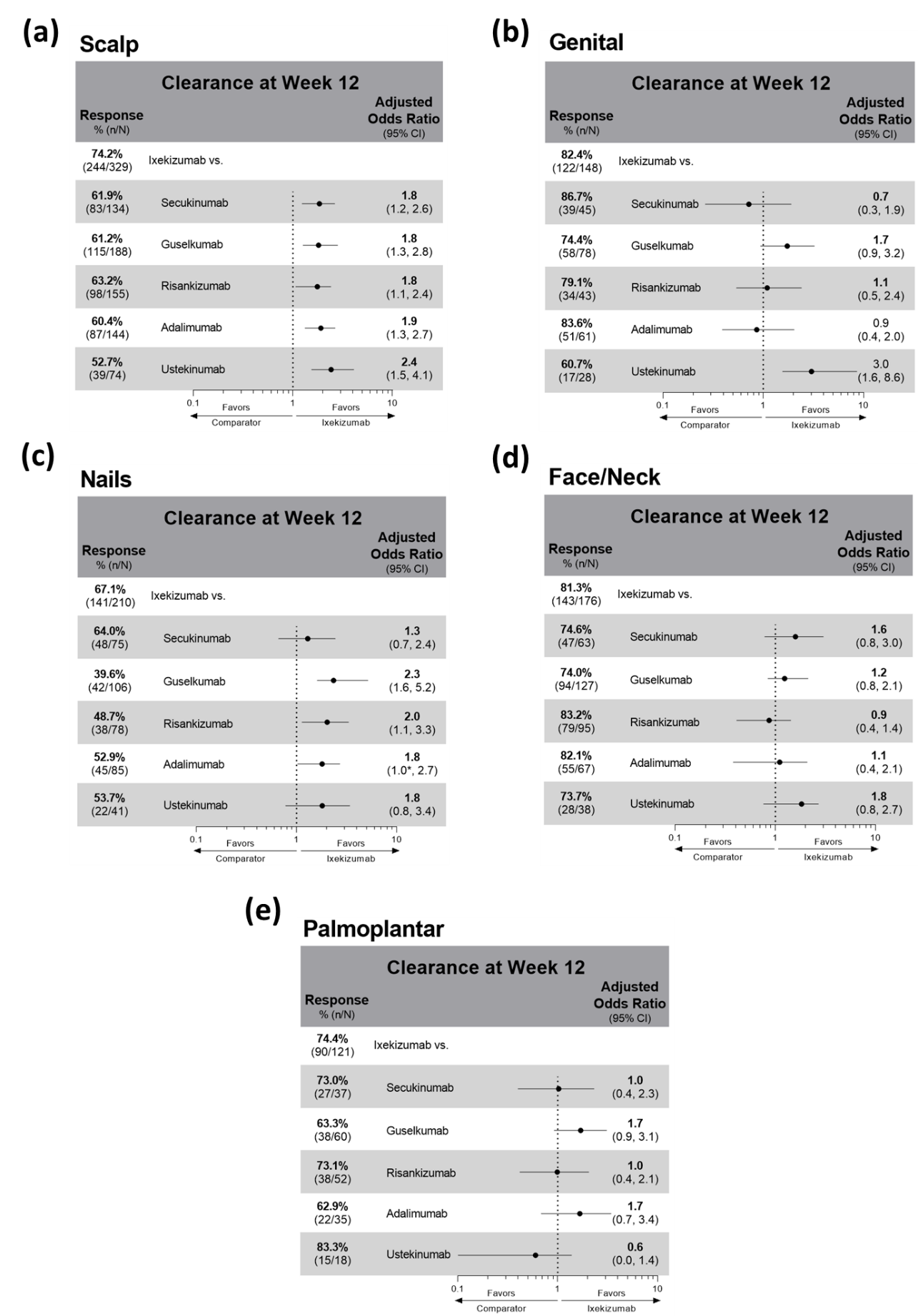


**Figure S2:** **Subgroup of patients who received the EMA-approved on-label dosing.** Unadjusted response rates and comparative adjusted odds ratios of ixekizumab versus individual treatments at for patients with baseline involvement and clearance at week 12 of **(a)** scalp psoriasis **(b)** genital psoriasis **(c)** nail psoriasis **(d)** face and/or neck psoriasis **(e)** palmoplantar psoriasis. Comparative results are statistically significant if 95% CIs of the odds ratios do not cover 1. ^*^Denotes that the result is significant as the lower CI is greater than 1.0. The lower CI for the ixekizumab vs. adalimumab odds ratio for nail clearance is 1.025. Missing data imputed as non-response. CI, confidence interval; n, number of patients with clearance of special area; N, number of patients in each treatment group.

**Supplementary Table 1:** Unadjusted response rates for clearance of special area at week 12 and confidence intervals across treatments for all patients. Numbers of patients with clearance in each treatment cohort and group are provided in Figures 2 and 3.

| **Clearance of**  **special area** | **Anti-IL-17A** | **Other Biologics** | **IXE** | **SEC** | **GUS** | **RIS** | **ADA** | **UST** |
| --- | --- | --- | --- | --- | --- | --- | --- | --- |
| Scalp | **70.4%**  (66.4, 74.5) | **61.5%**  (58.1, 64.8) | **74.1%**  (69.5, 78.7) | **61.9%**  (54.1, 69.8) | **61.1%** (54.4, 67.7) | **64.5%**  (57.4, 71.7) | **58.3%**  (51.2, 65.4) | **53.7%**  (42.9, 64.5) |
| Genital | **83.4%**  (78.3, 88.5) | **75.7%**  (70.9, 80.6) | **82.5%**  (76.5, 88.5) | **86.3%**  (76.8, 95.7) | **74.4%**  (64.9, 83.8) | **80.9%**  (69.6, 92.1) | **78.1%**  (68.6, 87.6) | **61.3%**  (44.1, 78.4) |
| Nails | **65.9%**  (60.6, 71.2) | **49.7%**  (45.0, 54.3) | **66.5%**  (60.3, 72.7) | **64.3%**  (54, 74.5) | **40.0%**  (31.0, 49.0) | **50.0%**  (39.6, 60.4) | **51.4%**  (41.9, 61) | **55.6%**  (41.0, 70.1) |
| Face and/or neck | **78.5%**  (73.6, 83.5) | **78.2%**  (74.5, 81.9) | **80.9%**  (75.2, 86.5) | **72.6%**  (62.4, 82.8) | **74.5%**  (67.3, 81.7) | **84.3%**  (77.3, 91.4) | **81.0%**  (72.6, 89.3) | **75.6%**  (62.5, 88.8) |
| Palms and/or soles | **73.6%**  (67, 80.1) | **71.1%**  (65.6, 76.5) | **73.3%**  (65.7, 80.9) | **74.4%**  (61.4, 87.5) | **64.6%**  (53, 76.2) | **70.7%**  (59, 82.4) | **65.4%**  (52.5, 78.3) | **78.9%**  (60.6, 97.3) |

Data reported as unadjusted CIs for percentage response rates. Data are NRI. ADA, adalimumab; CI, Confidence Interval; GUS, guselkumab; IL, interleukin; IXE, ixekizumab; NRI, non-responder imputation; RIS, risankizumab; SEC, secukinumab; UST, ustekinumab

**Supplementary Table 2:** Unadjusted response rates for clearance of special area at week 12 and confidence intervals across treatments for patients treated with EMA-approved on-label dosing. Number of patients with clearance in each treatment cohort and group are provided in Figures S1 and S2.

| **Clearance of**  **special area** | **Anti-IL-17A** | **Other Biologics** | **IXE** | **SEC** | **GUS** | **RIS** | **ADA** | **UST** |
| --- | --- | --- | --- | --- | --- | --- | --- | --- |
| Scalp | **70.6%**  (66.5, 74.8) | **62.1%**  (58.6, 65.7) | **74.2%**  (69.4, 78.9) | **61.9%**  (53.7, 70.2) | **61.2%**  (54.2, 68.1) | **63.2%**  (55.6, 70.8) | **60.4%**  (52.4, 68.4) | **52.7%**  (41.3, 64.1) |
| Genital | **83.4%**  (78.2, 88.7) | **76.5%**  (71.4, 81.6) | **82.4%**  (76.3, 88.6) | **86.7%**  (76.7, 96.6) | **74.4%**  (64.7, 84.0) | **79.1%**  (66.9, 91.2) | **83.6%**  (74.3, 92.9) | **60.7%**  (42.6, 78.8) |
| Nails | **66.3%**  (60.8, 71.8) | **50.4%**  (45.4, 55.3) | **67.1%**  (60.8, 73.5) | **64.0%**  (53.1, 74.9) | **39.6%**  (30.3, 48.9) | **48.7%**  (37.6, 59.8) | **52.9%**  (42.3, 63.6) | **53.7%**  (38.4, 68.9) |
| Face and/or neck | **79.5%**  (74.4, 84.6) | **77.9%**  (73.9, 81.9) | **81.3%**  (75.5, 87) | **74.6%**  (63.9, 85.4) | **74.0%**  (66.4, 81.6) | **83.2%**  (75.6, 90.7) | **82.1%**  (72.9, 91.3) | **73.7%**  (59.7, 87.7) |
| Palms and/or soles | **74.1%**  (67.2, 80.9) | **70.6%**  (64.7, 76.5) | **74.4%**  (66.6, 82.2) | **73.0%**  (58.7, 87.3) | **63.3%**  (51.1, 75.5) | **73.1%**  (61, 85.1) | **62.9%**  (46.8, 78.9) | **83.3%**  (66.1, 100.6) |

Data reported as unadjusted CIs for percentage response rates. Data are NRI. ADA, adalimumab; CI, Confidence Interval; GUS, guselkumab; IL, interleukin; IXE, ixekizumab; NRI, non-responder imputation; RIS, risankizumab; SEC, secukinumab; UST, ustekinumab
